# Alpha‐1‐antitrypsin deficiency (carrier) as possible risk factor for development of colonic diverticula. A multicentre prospective case–control study: the ALADDIN study

**DOI:** 10.1111/codi.15270

**Published:** 2020-09-01

**Authors:** S. J. Rottier, L. C. Dreuning, J. van Pelt, A. A. W. van Geloven, X. D. Y. Beele, P. M. Huisman, W. Y. Deurholt, C. A. Rottier, K. van Leeuwen, M. de Boer, G. van Mierlo, M. A. Boermeester, W. H. Schreurs, for the ALADDIN Collaborative Study Group

**Affiliations:** ^1^ Department of Surgery Northwest Clinics Alkmaar/Den Helder The Netherlands; ^2^ Department of Surgery Tergooi Hospital Hilversum The Netherlands; ^3^ Department of Surgery Academic Medical Center Amsterdam The Netherlands; ^4^ Department of Clinical Laboratory Northwest Clinics Alkmaar/Den Helder The Netherlands; ^5^ Department of Radiology Tergooi Hospital Hilversum The Netherlands; ^6^ Department of Radiology Northwest Clinics Alkmaar/Den Helder The Netherlands; ^7^ Department of Molecular and Cellular Hemostasis Sanquin Blood Supply Division Research and Landsteiner Laboratory of the Academic Medical Center University of Amsterdam Amsterdam The Netherlands; ^8^ Department of Immunopathology Sanquin Blood Supply Division Research and Landsteiner Laboratory of the Academic Medical Center University of Amsterdam, Amsterdam The Netherlands

**Keywords:** Alpha‐1‐antitrypsin, connective tissue disease, diverticula, diverticulitis, diverticulosis

## Abstract

**Aim:**

Connective tissue changes due to ageing or diseases leading to changes in the colonic wall are one theory for the development of diverticula. Alpha‐1‐antitrypsin (A1AT), a protease inhibitor that protects connective tissue, possibly plays a role in the aetiology of diverticulosis. The aim of this study was to explore associations between the development of diverticula and A1AT deficiency.

**Methods:**

This was a multicentre prospective case–control study. A total of 221 patients aged ≥ 60 years with acute abdominal pain undergoing abdominal CT were included and analysed. Patients with diverticula were defined as the research group, patients without diverticula as controls. Genotype analysis for A1AT deficiency was performed.

**Results:**

Twenty‐six of 221 (11.8%) patients were diagnosed with (being a carrier of) A1AT deficiency. A non‐significant difference in prevalence between patients with and without diverticula was found, 20 (13.9%) of 144 *vs* 6 (7.8%) of 77, respectively, with a crude OR of 1.9 (95% CI 0.7–5.0; *P* = 0.186) and after adjustment for confounders an adjusted OR of 1.5 (95% CI 0.5–4.0; *P* = 0.466). A non‐significant difference in 30‐day mortality rate from acute diverticulitis between A1AT deficient patients (or carriers) and those without was observed: two (22.2%) of nine patients with A1AT deficiency *vs* 1 (1.8%) of 55 without.

**Conclusion:**

We found no convincing evidence that A1AT deficiency plays a role in the aetiology of diverticulitis, although deficient patients and carriers had a higher mortality when experiencing diverticulitis. Diverticulitis is a multifactorial disease and larger numbers may be needed to explore the role of A1AT deficiency among other contributing factors.

**What does this paper add to the literature?**

Primarily in case reports, connective tissue diseases have been mentioned as a risk factor for diverticulitis. Furthermore, connective tissue changes due to ageing or diseases may lead to changes in the colonic wall and development of diverticula. The present study explores associations between connective tissue, diverticula and diverticulitis. This knowledge may open new possibilities for prevention or treatment.

## Introduction

The incidence of colonic diverticulitis increases with age and is primarily a disease amongst the western population. When searching the literature for possible risk factors for the development of diverticula, diet, obesity and alcohol consumption are often mentioned [1, 2, 3]. For the development of acute diverticulitis medication such as corticosteroids, smoking and hereditary factors are possible risk factors [4, 5, 6]. However, the true underlying pathophysiological mechanism that leads to the formation of colonic diverticula remains unclear. Common theories about the aetiology causing colonic diverticula are the influence of diet, specifically the lack of fibre in the western diet, and the microbiome theory [2, 7, 8].

In the published study protocol of the present study (ALADDIN study) [9], the hypothesis of the onset of diverticula being the result of an age‐related disorder was described in further detail. In summary, ageing eventually leads to alteration of the colonic epithelium which induces a decrease in colonic wall strength, partly due to changes in the collagen structure [10, 11]. A pathology study found that colonic collagen from subjects affected by colonic diverticulosis had a higher number of crosslinks than subjects with unaffected colonic tissue, illustrating that these structural changes have a greater impact than the changes as part of the natural ageing process [12]. Also, an increase of the less stable collagen type 3 combined with increased concentrations of tissue‐degrading matrix metalloproteinases (MMPs) and their inhibitor (TIMP‐1) was observed in patients with (complicated) diverticular disease [13, 14, 15]. This supports the hypothesis that colonic diverticulosis could be the result of an exaggerated and premature ageing process caused by changes of collagen structure of the colonic wall. One specific feature that could play a role in the development of diverticula is alpha‐1‐antitrypsin (A1AT) deficiency, a hereditary disorder which affects individuals of all racial subgroups worldwide. In Europe, the prevalence of A1AT deficiency is 0.24%, whereas the prevalence of A1AT carriers is approximately 8% [16]. A1AT is a protease inhibitor which protects the connective tissue of the lungs when elastase is released [17, 18]. Collagen types I and III give the alveolar wall of the lungs its structural form; MMPs that are involved in tissue repair and remodelling try to maintain this wall structure [19, 20, 21]. Some of the same MMPs involved in connective tissue metabolism are altered in patients with lung emphysema as well as in patients with (complicated) diverticular disease [21, 22]. In patients with A1AT deficiency, specifically individuals with type PiZZ, mortality was increased due to respiratory and hepatic disease and pulmonary embolism, compared to a population without this pathological allele for A1AT, as expected. Patients with this deficiency also had a higher mortality due to complicated colonic diverticulitis, which supports the hypothesis that A1AT could be associated with the development of diverticula and (complicated) diverticulitis [23].

## Method

### Study design and population

The trial protocol (rationale, design and population) has been published [9]. Here, we summarize the most important details. In this multicentre, prospective, case–control study (ALADDIN study) patients were included from three non‐academic hospitals in the Netherlands. All patients ≥ 60 years with acute abdominal pain who had undergone an abdominal CT were recruited from the Emergency Department of each participating hospital. Patients were eligible for the research group when the abdominal CT revealed > 5 diverticula. Patients without diverticula, defined as 0 to ≤ 5 diverticula on abdominal CT, were included in the control group. The CTs were reviewed by radiologists who reported the diagnosis, the number of diverticula, the location of diverticula and, in the case of diverticulitis, the Hinchey classification. All patients eligible for this study were screened by the attending emergency physician, and informed consent was taken. The patient file and a patient questionnaire aimed to identify known risk factors for developing diverticula or acute diverticulitis. This questionnaire provided information on family history, patient diet, alcohol intake, packyears, physical exercise, stool production, and pattern and use of anticoagulants, nonsteroidal anti‐inflammatory drugs (NSAIDs) and/or immunosuppressants. Blood samples were collected and the concentration of A1AT in serum was determined by immunoassay. Genotype analysis was performed, which revealed whether an individual had a normal genotype (PiMM), was a carrier of A1AT deficiency (PiM Heerlen, PiMS, PiMZ or PiMF) or had A1AT deficiency (PiSS, PiSZ and PiZZ).

### Study objective

The objective was to determine the prevalence of A1AT deficiency (carriers) in patients with and without colonic diverticula.

### Laboratory analysis

#### DNA extraction, whole genome amplification

DNA was extracted from 600 µl plasma or from 200 µl buffycoat. The Qiagen Mini Blood Kit (Qiagen Benelux BV, Venlo, The Netherlands) was used for the extraction of DNA. In some cases where the DNA concentration was too low to obtain the minimal coverage criteria, whole genome amplification was performed on DNA extracted from a new plasma sample. The Qiagen REPLI‐g FFPE kit (Qiagen Benelux BV) was used for whole genome amplification.

#### Sequencing

A targeted custom AmpliSeq panel was designed containing coding sequences for three known A1AT deficiency causing genes (SERPINA1, SERPINA3 and ELA2). In total 27 amplicons were designed covering 6.5 kb. The Ion Chef (Thermo Fisher Scientific, Waltham, Massachusetts, USA) was used to create a next‐generation sequencing library for each sample. These libraries were pooled on an Ion chip and sequenced on an Ion S5 sequencer (Thermo Fisher Scientific). Using a hotspot file on torrent suite 5.4 or 5.8 software (Thermo Fisher Scientific) known variants responsible for A1AT deficiency were called.

### Outcome measures

The primary study parameter was prevalence of A1AT deficiency or carriers in patients with and without colonic diverticula. Secondary study parameters were previous episodes of diverticulitis in patients with and without (carrier alleles for) A1AT deficiency and the number of diverticulitis‐related hospital admissions and diverticulitis‐related complications in patients with and without (carrier alleles for) A1AT deficiency.

### Ethical considerations

The study was conducted in accordance with the principles of the Declaration of Fortaleza and ‘good clinical practice’ guidelines. The protocol (version 4, amendment 4, 7 December 2018) was approved by the Medical Research Ethics Committees of VU University Medical Center (MREcVUmc). Consent was also obtained from the participating centres. The patients were counselled and written informed consent was obtained from all patients if inclusion criteria were met.

### Statistical analysis

Nominal and ordinal variables were described as numbers with proportions. Continuous variables were described as means with standard deviation (SD) or medians with interquartile range (IQR). Between group (cases *vs* controls) differences with respect to nominal and ordinal variables were analysed using the chi‐squared test or the Fisher exact test where appropriate. Differences with respect to continuous variables were analysed using the independent samples *t* test or the Mann–Whitney *U* test depending on distribution. The strength of the association between exposure of A1AT pathology and the occurrence of diverticulosis was analysed by means of logistic regression analysis and expressed as an OR with 95% CI. Multivariable logistic regression analysis was used to correct this association for confounding effects. All secondary study parameters (except for A1AT concentration) were potential confounders and were selected as such in a forward selection procedure with a limit of 10% change in effect size using a basic logistic regression model with only exposure of A1AT pathology as independent variable and diverticulosis as dependent variable. The confounder with the largest change in effect size of the determinant (exposure of A1AT pathology) was included in the new model. The selection procedure was then repeated on the new model, using the remaining covariables. The procedure was stopped after none of the covariables changed the effect size by more than 10% or after reaching the maximum number of confounders defined as 10% of the number of patients with diverticulosis. Multiple imputation was used to deal with missing data. All secondary study parameters were analysed in patients with diverticula and compared between patients with and without the A1AT deficiency (carriers). Correction for multiple testing was done by using the Bonferroni correction. Differences with respect to continuous variables were analysed using the independent samples *t* test or Mann–Whitney *U* test depending on the distribution. Differences with regard to nominal or ordinal variables were analysed using the chi‐squared test or Fisher exact test where appropriate.

STROBE guidelines for reporting will be followed [24]. All analyses were performed using spss, version 24.0 (SPSS Inc., Chicago, Illinois, USA).

### Sample size calculation

It was assumed that there are at least 116 million carriers (PiMS and PiMZ) and 3.4 million deficiency allele combinations (PiSS, PiSZ and PiZZ) worldwide. The prevalence of A1AT deficiency carriers in Europe was calculated to be approximately 8% [16]. No previous information on the prevalence of A1AT deficiency (carriers) in patients with or without diverticula existed. Therefore, we could only speculate on the possible difference in prevalence between these two groups. To assess a 10% difference in prevalence at a significance level of 5% and a power of 80%, 115 patients per group were needed. Since the literature indicated that the prevalence of diverticulosis was around 50%, inclusion of cases and controls was expected to progress equally [25, 26].

### Data collection and monitoring

Data were collected from the medical records of the patients. Additional information was gathered using a questionnaire.

## Results

### Study design and population

The initial plan was to perform phenotype analyses because genotype analyses were (financially) not possible. However, during the inclusion period new developments in genotype analysis became available allowing us to perform genotype analysis. At that time, over 75% of the patients were already included, from whom we collected plasma samples.

**Figure 1:**
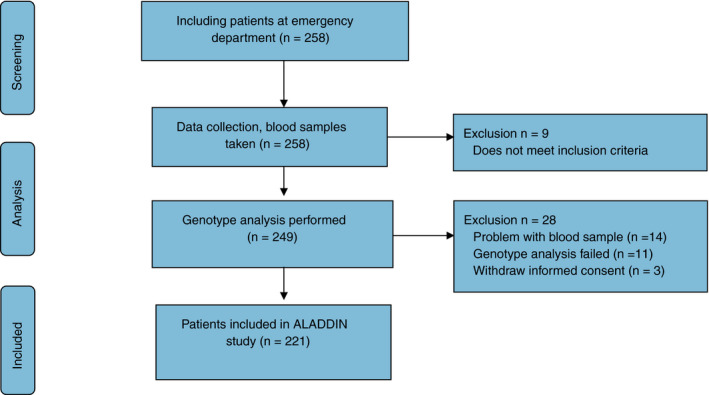
The ALADDIN study flowchart comprising the study population, including inclusion and exclusion criteria.

A total of 258 patients were included; nine patients were excluded because of incomplete colon or free air on abdominal CT, making a reliable count of diverticula impossible. For analyses, 249 patients were included. Genotype analysis is possible from plasma samples but such samples contain limited amounts of DNA. For this reason, genotype analysis was not possible in 25 patients. Three patients withdrew informed consent after analyses were done, leaving a total of 221 patients (Fig. 1). The progression of inclusion of diverticulosis patients and controls did not proceed at the same pace. The final patient group with five or more diverticula comprised 144 (65.2%) patients and the control group 77 (34.8%) patients without at least five diverticula. The groups were comparable for age, gender and comorbidity such as cardiovascular diseases, diabetes mellitus and pulmonary pathology. Differences were observed for body mass index (*P* = 0.012). Possible risk factors extracted from patient records and questionnaires showed differences between the groups only for a positive family history of diverticulitis (*P* = 0.019) and use of NSAIDs (*P* = 0.038; Table 1). Patients were diagnosed with acute diverticulitis, appendicitis, cholecystitis, pancreatitis, obstructive ileus, malignancy, stomach or bowl perforation, ischaemia of the intestine, vascular disease, colitis, cardiac or pulmonary pathology, urological pathology, gastro‐enteritis and obstipation.

**Table 1:** Baseline characteristics including possible risk factors for diverticulosis or diverticulitis based on the literature.

	All patients (*N* = 221)	Diverticula group (*N* = 144)	Control group (*N* = 77)	*P* value
Age, years, mean (SD)	72.28 (7.94)	72.88 (8.37)	71.16 (6.96)	0.105
Female gender, *n* (%)	128 (57.9)	82 (56.9)	46 (59.7)	0.688
Nationality, Dutch, *n* (%)† ^,^ ‡	213 (98.2)	142 (99.3)	71 (95.9)	0.116
BMI (kg/m^2^), mean (SD)	26.78 (4.92)	27.38 (5.07)	25.64 (4.44)	**0.012**
Comorbidity, *n* (%)				
Myocardial infarction	26 (11.8)	15 (10.4)	11 (14.3)	0.395
Cerebral ischaemia	20 (9.0)	11 (7.6)	9 (11.7)	0.317
Hypertension	79 (35.7)	55 (38.2)	24 (31.2)	0.299
Heart failure†	10 (4.5)	7 (4.9)	3 (3.9)	1.000
Peripheral arterial disease	16 (7.2)	11 (7.6)	5 (6.5)	0.754
Diabetes mellitus	32 (14.5)	20 (13.9)	12 (15.6)	0.733
Renal failure†	8 (3.6)	6 (4.2)	2 (2.6)	0.717
Pulmonary diseases	56 (25.3)	39 (27.1)	17 (22.1)	0.415
Connective tissue disease	32 (14.5)	22 (15.3)	10 (13.0)	0.645
Family members with diverticulitis, *n* (%)§	19 (9.1)	17 (12.5)	2 (2.7)	**0.019**
Fibre diet, *n* (%)* ^,^ ¶	109 (50.9)	67 (47.9)	42 (56.8)	0.215
Fluid intake ≤ 1.5 l/day, *n* (%)**	55 (25.6)	30 (21.4)	25 (33.3)	0.057
Coffee intake, *n* (%)† ^,^ **	206 (95.8)	133 (95.0)	73 (97.3)	0.501
Alcohol intake, *n* (%)††	149 (68.3)	98 (68.5)	51 (68.0)	0.936
Current smoker, *n* (%)‡‡	34 (15.5)	21 (14.7)	13 (17.1)	0.638
Packyears, median (IQR)††	6 (0–26)	7 (0–26)	4 (0–27)	0.625
Physical exercise> 30 min/day, *n* (%)**	56 (26.0)	41 (29.3)	15 (20.0)	0.139
Daily stool production, *n* (%)§	181 (86.6)	118 (86.1)	63 (87.5)	0.783
Changes in defaecation pattern, *n* (%)§§	60 (28.8)	40 (29.6)	20 (27.4)	0.734
Use of anticoagulants, *n* (%)^¶¶^	94 (42.5)	63 (43.8)	31 (40.3)	0.617
Use of NSAIDs, *n* (%)***	46 (21.7)	24 (17.4)	22 (29.7)	**0.038**
Use of immunosuppressants, *n* (%)† ^,^ †††	13 (5.9)	10 (6.9)	3 (3.9)	0.550
Average no. of diverticula	6–10	11–15	0–5	–

BMI, body mass index; IQR, interquartile range; NSAIDs, nonsteroidal anti‐inflammatory drugs.

Bold indicates statistically significant values.

* According to the Dutch Nutrition Centre (Voedingscentrum).

† Fisher’s exact test.

‡ Eight missing.

§ Twelve missing.

¶ Seven missing.

** Six missing.

†† Three missing.

^‡‡^ Two missing.

§§ Thirteen missing.

¶¶ Acetylsalicylic acid, vitamin‐K antagonists or heparin.

*** Nine missing.

††† One missing.

### Prevalence of A1AT deficiency (carriers)

In the overall population, two patients were diagnosed with A1AT deficiency (one with PiZZ and one with PiSS) and 24 patients were diagnosed as being a carrier of A1AT deficiency (Table S1). In the diverticulosis group, A1AT deficiency (or carrier) was found in 20 (13.9%) of 144 patients, whereas in the control group this was found in six (7.8%) of 77 patients. This difference was notable but not significant: crude OR of 1.9 (95% CI 0.732–4.974), *P* = 0.186. After adjustment for confounders (body mass index, use of NSAIDs, use of corticosteroids, nationality and fluid intake ≤ 1.5 l/day) the adjusted OR for development of diverticula in patients with, or who are carriers for, A1AT deficiency was 1.5 (95% CI 0.5–4.0; *P* = 0.466; Table 2).

**Table 2:** Crude odds ratio and adjusted odds ratio with *P* values for A1AT deficiency (carrier) as a risk factor for diverticulosis, acute diverticulitis and a more severe course of acute diverticulitis.

	All patients (*N* = 221)	Diverticula group (*N* = 144)	Control group (*N* = 77)	Crude OR (95% CI)	*P* value	Adjusted OR (95% CI)	Adjusted *P* value
A1AT deficiency (carriers)	26 (11.8)	20 (13.9)	6 (7.8)	1.909 (0.732–4.974)	0.186	1.455 (0.532–3.981)	0.466

A1AT, alpha‐1‐antitrypsin.

### Subgroup analysis of patients with diverticula

Among the research group of 144 patients with diverticula, 64 patients were diagnosed with acute diverticulitis at first presentation. The number of patients with acute diverticulitis at presentation (*P* = 0.957), number of diverticula (*P* = 0.705) or location of diverticula (*P* = 0.150) were comparable among patients with and without A1AT deficiency (carriers) (Table 3a).

**Table 3:** (a) Baseline characteristics for patients with diverticulosis or diverticulitis comparing patients with and without A1AT deficiency (carriers); (b) baseline characteristics for patients with acute diverticulitis comparing patients with and without A1AT deficiency (carriers).

(a)				
	Diverticula group (*N* = 144)	With A1AT carrier/deficiency (*N* = 20)	Without A1AT carrier/deficiency (*N* = 124)	*P* value^‡^
Number of diverticula, *n* (%)*				
6–10 diverticula	25 (17.7)	4 (20.0)	21 (17.4)	0.705
11–15 diverticula	22 (15.6)	3 (15.0)	19 (15.7)	
16–20 diverticula	18 (12.8)	3 (15.0)	15 (12.4)	
21–25 diverticula	17 (12.1)	4 (20.0)	13 (10.7)	
> 25 diverticula	59 (41.8)	6 (30.0)	53 (43.8)	
Location of diverticula, *n* (%)*				
Left	95 (67.4)	17 (85.0)	78 (64.5)	0.150
Right	0	0	0	
Both	35 (24.8)	3 (15.0)	32 (26.4)	
Pan	11 (7.8)	0	11 (9.1)	
Diverticulitis at presentation, *n* (%)	64 (44.8)	9 (45.0)	55 (44.4)	0.957

(b)				
	Patients with acute diverticulitis (*N* = 64)	With A1AT carrier/deficiency (*N* = 9)	Without A1AT carrier/deficiency (*N* = 55)	*P* value^‡^
Previous episode of acute diverticulitis, *n* (%)* ^,^ † ^,^ ‡	20 (32.3%)	4 (44.4)	16 (30.2)	0.453
Hinchey classification, *n* (%)*				
1a (uncomplicated)	44 (68.8)	5 (55.6)	39 (70.9)	0.443
> 1b (complicated)	20 (31.3)	4 (44.4)	16 (29.1)	
C‐reactive protein (CRP) at presentation, median (IQR)§	108 (54–188)	95 (35–210)	108 (54–188)	0.796
White blood cell count (WBC), leukocytes at presentation, mean (SD)§	13.7 (7.2)	15.1 (5.0)	13.5 (7.5)	0.571
Days of admission, median (IQR)	3 (0–7)	1 (0–12)	3 (0–7)	1.000
Intervention needed, *n* (%)* ^,^ ‡ ^,^ §	7 (11.1)	1 (11.1)	6 (11.1)	1.000
Received antibiotic treatment, *n* (%)*	29 (45.3)	3 (33.3)	26 (47.3)	0.494
Emergency surgery, *n* (%)*	9 (14.1)	1 (11.1)	8 (14.5)	1.000
ICU admission, *n* (%)*	3 (4.7)	0 (0)	3 (5.5)	1.000
30‐day mortality, *n* (%)*	3 (4.7)	2 (22.2)	1 (1.8)	**0.0495**
Concentration A1AT, mean (SD)	2.17 (0.754)	1.80 (0.67)	2.23 (0.75)	0.111

A1AT, alpha‐1‐antitrypsin; ICU, intensive care unit; IQR, interquartile range.

Bold indicates statistically significant values.

* Fisher's Exact test.

† Two missing.

‡ Corrected for multiple testing (Bonferroni), significance level 0.05/11 = 0.0045.

§ One missing.

### Subgroup analysis of patients with acute diverticulitis

Sixty‐four of 221 patients were diagnosed with acute diverticulitis; nine of these 64 patients also had A1AT deficiency or were a carrier, and 55 of 64 patients were not. All parameters for disease severity were comparable between the two subgroups, such as Hinchey classification [uncomplicated diverticulitis (Hinchey 1a) *vs* complicated diverticulitis (Hinchey 1b, 2, 3)], C‐reactive protein level and leukocyte count at presentation, days of hospital admission, whether a radiological or surgical intervention was needed, antibiotic treatment, need for intensive care unit admission, and 30‐day mortality rate. A non‐significant difference in 30‐day mortality rate from acute diverticulitis between A1AT deficient patients (or carriers) and those without was observed: two (22.2%) of nine patients with A1AT deficiency *vs* one (1.8%) of 55 without (Table 3b). No difference was found in the number of previous episodes of acute diverticulitis (Table 3b).

## Discussion and conclusions

The hypothesis of the present study was that connective tissue diseases, in particular A1AT deficiency or being a carrier of this deficiency, contribute to the development of diverticula. The results showed a non‐significant difference in the prevalence of A1AT between patients with and without diverticula, 13.9% *vs* 7.8% respectively.

Because the prevalence of A1AT deficiency is low, approximately 0.24% in Europe, focus was on finding A1AT deficient carriers, which has a prevalence of 8% according to the literature [16]. We did anticipate that the true prevalence of A1AT deficiency (carriers) may be underestimated. Indeed, we found a higher prevalence in the study population, being 11.8%. While assessing all other known risk factors for developing diverticulosis and diverticulitis the present study gives a good impression of the true influence of connective tissue disease related A1AT deficiency or carrier in the aetiology of diverticulosis. The current literature often mentions that connective tissue diseases play a role in diverticulitis; however, most of the information is based on old data, case reports and twin studies [27, 28, 29]. The results of the subgroup of patients with acute diverticulitis were notable. The deficient group was non‐significantly more often diagnosed with complicated diverticulitis (>Hinchey 1b) (44.4% *vs* 29.1%) than the non‐deficient group. Thirty‐day mortality was non‐significantly higher in the deficient group (22.2% *vs* 1.8% without A1AT deficiency; Table 3b). Other than before the start of this study, A1AT deficiency may play a role in disease severity (course of disease) rather than development of diverticulitis, or both.

A limitation of this study was the fact that our laboratory analysis was changed during the study from phenotype analysis to genotype analysis. Although genotype analysis is a better method for diagnosing a patient with A1AT deficiency (or being a carrier), plasma samples – which were being collected at that point – contained limited amounts of DNA. For this reason, genotype analysis was not possible in 25 patients. Another limitation is that the literature indicates that the prevalence of diverticulosis in symptomatic patients is around 50% based on CT colonography [25, 26]. Based on these data, inclusion of diverticula cases and controls was expected to progress equally. However, in the ALADDIN study, 144 (65.2%) patients were assigned to the research group and 78 (34.8%) patients to the control group, screened between 2017 and 2019 by abdominal CT. A possible explanation for the asymmetric distribution of cases and controls in the ALADDIN study would be that the study involved a population with acute abdominal pain instead of symptomatic non‐acute patients. This may have caused the study to be underpowered for the hypothesis. Moreover, we did not perform a separate sample size calculation for the sub‐analysis of diverticulitis patients only, but the subgroup analysis itself was pre‐specified in the protocol.

At the time this study was conducted little or no evidence was available in the literature on the role of connective tissue diseases in the development of diverticula. Recently, a genome‐wide association analysis of diverticular disease and connective tissue pathological mechanisms has been published [30]. Regarding the polygenetic risk signature of diverticular disease, an overlap with syndromatic neuromuscular, connective tissue and morphogenesis disorders and previous findings was observed. Manifestation of diverticulitis may be triggered by epithelial dysfunction of an altered colon anatomy [30]. This is in line with our hypothesis that changes in anatomy, specifically the connective tissue, play a role in the development of diverticula and possibly are involved in acute diverticulitis.

In this study, we found no convincing evidence that A1AT deficiency (or carrier status) plays a role in the aetiology of diverticulitis, although deficient patients and carriers had a higher mortality when experiencing diverticulitis. The formation of diverticula and the development of acute diverticulitis seem to be multifactorial. We believe that our hypothesis cannot be rejected permanently or firmly based on the observed but non‐significant differences in prevalence. Patients who have (or are carriers for) A1AT deficiency showed a trend towards more diverticula and, when diagnosed with acute diverticulitis, appeared to have a more severe course of diverticulitis compared to the patients with a normal type of A1AT. Foremost, these results indicate that further research is justified as a trend but no convincing evidence was found that A1AT deficiency plays a role in the aetiology of diverticulitis. This may be due to the fact that diverticulitis is a multifactorial disease and larger numbers may be needed to explore the role of A1AT deficiency amongst other contributing factors. Future studies not only may focus on A1AT deficiency alone but may extend to multiple connective tissue diseases.

## Conflicts of interest

Drs S.J. Rottier, Drs L.C. Dreuning, Dr J. van Pelt, Dr A.A.W. van Geloven, Drs X.Y.D. Beele, Drs P.M. Huisman, Drs W.Y. Deurholt, Drs C.A. Rottier, K. van Leeuwen, M. de Boer, G. van Mierlo, Professor M.A. Boermeester, Dr W.H. Schreurs have no conflicts of interest or financial ties to disclose related to the study subject. The ALADDIN study received no specific grant from any funding agency in the public or commercial sectors. We did receive local funding from the participating local hospitals (Northwest Clinics, location Alkmaar, The Netherlands, and Tergooi, Hilversum, The Netherlands) to pay for the genotype analysis and salary of the PhD student.

## Author contributions

Drs S.J. Rottier was responsible for the conception, design, acquisition of data, analysis and interpretation of data. Drs L.C. Dreuning was responsible for acquisition of data. Dr J. van Pelt, Dr A.A.W. van Geloven, Drs X.Y.D. Beele, Drs P.M. Huisman, Drs W.Y. Deurholt, Drs C.A. Rottier, K. van Leeuwen, M. de Boer, G. van Mierlo, Professor M.A. Boermeester, Dr W.H. Schreurs made substantial contribution to the conception, design and interpretation of data. Simone Rottier drafted the manuscript. Drs L.C. Dreuning, Dr J. van Pelt, Dr A.A.W. van Geloven, Drs X.Y.D. Beele, Drs P.M. Huisman, Drs W.Y. Deurholt, Drs C.A. Rottier, K. van Leeuwen, M. de Boer, G. van Mierlo, Professor M.A. Boermeester, Dr W.H. Schreurs critically revised the manuscript for important intellectual content. Drs S.J. Rottier, Drs L.C. Dreuning, Dr J. van Pelt, Dr A.A.W. van Geloven, Drs X.Y.D. Beele, Drs P.M. Huisman, Drs W.Y. Deurholt, Drs C.A. Rottier, K. van Leeuwen, M. de Boer, G. van Mierlo, Professor M.A. Boermeester, Dr W.H. Schreurs gave final approval of the version to be published and are accountable for all aspects of the work.

## Supporting information


**Figure S1.** Histogram with additional information on number of diverticula in the control and diverticula groups.
**Table S1.** Additional information on patients with A1AT deficiency (or carriers).
