# Supplementary material for: Alpha‐1‐antitrypsin deficiency (carrier) as possible risk factor for development of colonic diverticula. A multicentre prospective case–control study: the ALADDIN study
Source: Colorectal Dis. 2020 Sep 1;22(12):2243–51. doi: 10.1111/codi.15270 (PMC7818100; doi:10.1111/codi.15270)
Supplement: Supplementary file 1 — Figure S1. Histogram with additional information on number of diverticula in the control and diverticula groups. Table S1. Additional information on patients with A1AT deficiency (or carriers). [file CODI-22-2243-s001.docx]

**SUPPLEMENTARY MATERIAL**

Figure 1 Histogram with additional information on number of diverticula in control and diverticula group


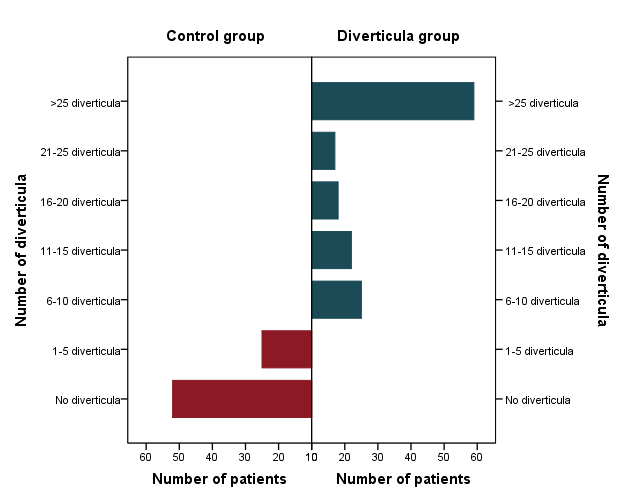


Table 1 Additional information on patients with A1AT deficiency (or carriers)

| **Variant name** | **Locus (hg19)** | **Reference** | **Genotype** | **Coding sequence**  **(**[**NM_000295.4**](http://www.ncbi.nlm.nih.gov/entrez/viewer.fcgi?db=nucleotide&id=NM_000295.4)**)** | **Protein** | **Number of patients** | **Case** | **Control** |
| --- | --- | --- | --- | --- | --- | --- | --- | --- |
| PiZZ | chr14:94844947 | C | T/T | c.1096G>A | p.(E366K) | 1 | 1 | 0 |
| PiMZ | chr14:94844947 | C | C/T |  |  | 10 | 7 | 3 |
| PiSS | chr14:94847262 | T | A/A | c.863A>T | p.(E288V) | 1 | 1 | 0 |
| PiMS | chr14:94847262 | T | T/A |  |  | 10 | 7 | 3 |
| PiM Heerlen | chr14:94844865 | G | G/A | c.1178C>T | p.(P393L) | 1 | 1 | 0 |
| PiMF | chr14:94847386 | G | G/A | c.739C>T | p.(R247C) | 3 | 3 | 0 |

* All variants were located on SERPINA 1 gene.
